# Semicentennial of Human Babesiosis, Nantucket Island

**DOI:** 10.3390/pathogens10091159

**Published:** 2021-09-09

**Authors:** Sam R. Telford, Heidi K. Goethert, Timothy J. Lepore

**Affiliations:** 1 Department of Infectious Disease and Global Health, Tufts University, 200 Westboro Road, North Grafton, MA 01536, USA; heidi.goethert@tufts.edu; 2 Nantucket Cottage Hospital, Nantucket, MA 02554, USA; tjalepore@me.com

**Keywords:** *Babesia microti*, human babesiosis, Nantucket Island, epidemiology, ecology, human risk

## Abstract

Fifty years ago, the index case of human babesiosis due to *Babesia microti* was diagnosed in a summer resident of Nantucket Island. Human babesiosis, once called “Nantucket fever” due to its seeming restriction to Nantucket and the terminal moraine islands of southern New England, has emerged across the northeastern United States to commonly infect people wherever Lyme disease is endemic. We review the history of babesiosis on Nantucket, analyze its epidemiology and ecology there, provide summaries of the first case histories, and comment on its future public health burden.

## 1. Introduction

Fifty years ago, a New England Journal of Medicine [1] case report summarized the index case of human babesiosis due to *Babesia microti*. Babesiosis had previously been reported in 4 patients (1 from Yugoslavia, 2 from Ireland, 1 from California), all of whom had been splenectomized; the Yugoslavian and Irish cases were due to *B. divergens*, a cattle parasite, and the California case was due to an unidentified *Babesia* sp. (likely *B. duncani*). The new case was in a spleen-intact person. Through 1976, 14 cases of symptomatic *B. microti* babesiosis had been identified on Nantucket and the infection was given the popular name “Nantucket fever” [2], even though sporadic cases were reported from nearby Martha’s Vineyard, Shelter Island, NY and Montauk, NY by 1977. From 2011–2015, 27 states reported 7612 cases of babesiosis, but only 7 states accounted for 95% of these (Massachusetts, New York, Connecticut, New Jersey, Rhode Island, Wisconsin and Minnesota [3]. Nantucket County still reports more cases of babesiosis than any other in the U.S., with annual incidence of >100 per 100,000 (compared to about 1 per 100,000 nationally). We revisit the early investigations of Nantucket fever in the 1970s and highlight the major findings that defined the epidemiology and ecology of this infection.

## 2. The Grey Lady

**Figure 1:**
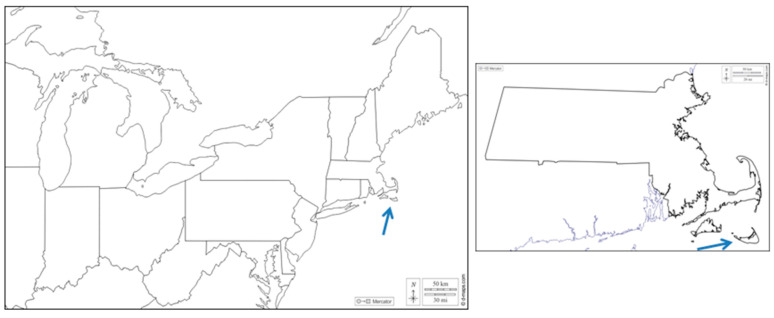
Nantucket Island, Massachusetts. Left panel, northeastern United States with arrow pointing to Nantucket Island. Right panel, Massachusetts with the location of Nantucket (arrow). Map from https://d-maps.com, accessed on 30 July 2021.

Nantucket Island is a 120 sq km island (land area; another 670 sq km is coastal waters) 50 km south of Cape Cod, Massachusetts (Figure 1). It was formed by the deposition of moraine from the retreat of the Laurentide ice sheet 15,000–18,000 years ago; the moraine and outwash deposits became an island due to rising sea levels 5000–6000 years ago. Due to the influence of strong winds containing oceanic salt spray, trees were limited to species that were relatively small and salt tolerant. European settlers were established by 1660, bringing cattle, horses, sheep and pigs. By the late 1700s, the island had become essentially treeless pasture, with a peak sheep herd of 15,000 (125/sq km). From the late 1600s to 1850, Nantucket’s economy was a function of whaling; Nantucket figures prominently in Melville’s Moby Dick. After the Civil War, a tourism industry developed, with an intensification by the 1920s.

**Figure 2:**
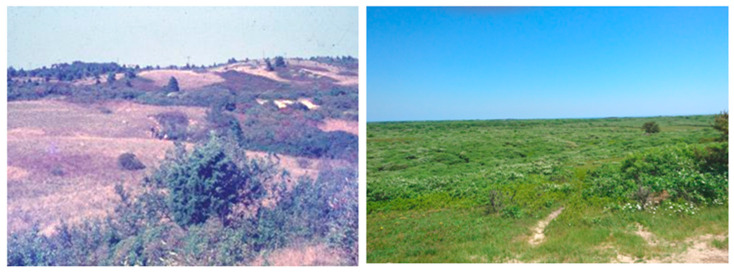
Changes in the landscape. Left panel, middle moors near Altar Rock, mid 1980s. “Heath” patches comprising grasses and low-lying shrubs, with interspersed thickets. Right panel, similar vantage point, 2021, demonstrating expansion of brush thicket into the heath.

The end of sheep pasturing allowed for successional growth of shrubs. The landscape is dominated by patches of thick scrub comprising bayberry (*Myrica pensylvanica*), saltspray rose (*Rosa rugosa*), poison ivy (*Rhus rhadicans*), black huckleberry (*Gaylussacia baccata*), highbush blueberry (*Vaccinium corymbosum*), sweet pepperbush (*Clethra alnifolia*), sumac (*Rhus glabra*), and scrub oak (*Quercus ilicifolia*). These patches are interspersed with grassland, although in most places scrub patches have expanded over the years (Figure 2). A pitch pine forest comprises 47 hectares in the center of the island. The climate is moderated by the Atlantic Ocean, with an average annual temperature of 10.5 °C (range of −3 °C in January to 23.9 °C in July) and 33.3 mm of rainfall. The island is often covered with a dense fog (hence the name “Grey Lady”).

Nantucket Island is accessible only by ferry or other boat, or by airplane. There are currently 17,200 year round residents, with as many as an additional 11,000 living there for 2 or more weeks during the tourist summer season (Memorial Day to Labor Day). There were 6600 seasonal workers in July 2017, and 500,000 visitor trips, 100,000 in August alone (www.nantucketdataplatform.com/projects, accessed on 30 July 2021). The median home value was $2.55 million and the average was $3.37 million at the end of 2020 and there are 12,675 housing units, 70.5% of which are owner occupied. There were 3713 households recorded in 2015–2019, with a median household income of $107,717.

The mammalian fauna includes no mesomammals (raccoons, skunks, weasels, fox, coyote, opossum) but has rodents and shrews typical for southern New England, with the exception of the red backed vole, *Myodes gapperi*. Norway rats (*Rattus norvegicus*) may be found in all habitats. There are large numbers of eastern cottontail rabbits (*Sylvilagus floridanus*). White tailed deer (*Odocoileus virginianus*) may be as dense as 56–65 per sq km. Besides the deer tick (*Ixodes dammini*, the junior subjective synonym for *I. scapularis*), there are American dog ticks, *Dermacentor variabilis* (increasingly scarce due to the use of preventives on domestic dogs; there are no other reproductive hosts for this tick there); rabbit ticks *I. dentatus* and *Haemaphysalis leporispalustris*, and an emerging infestation of Lone Star ticks, *Amblyomma americanum*.

None of these physical or socioeconomic characteristics, except perhaps the moderate climate, help explain why Nantucket was first recognized as and still is the most intensely zoonotic site for babesiosis due to *Babesia microti*.

## 3. Index and Other Early Cases

The first cases of Nantucket fever provided the general details of the course of illness and its management, as well as parasite strains that have been widely used in laboratory studies of the pathobiology of *B. microti* babesiosis. CDC investigations provided a good knowledge of the epidemiology and the asymptomatic to symptomatic ratio. CDC and Harvard studies outlined the general ecology of the parasite.

On 4 July 1969, a 59 year old woman who was a summer resident of Nantucket Island became ill and during the following 9 days sustained intermittent fever. The diagnosis remained elusive on Nantucket, but the critical observation, that she might have malaria, was made at St. Peter’s Hospital (SPH) in New Jersey. Diverse accounts (including [1,4]) have been provided about the connection between the Nantucket patient and New Jersey, but the true story appears in [5]: a friend of the index patient had asked a colorectal surgeon from SPH, Benjamin Glasser, who had treated members of the family, to come to Nantucket to see the “dying” patient, who was transported by private plane to SPH and admitted on 13 July. Malaria parasites were found by a microscopist examining a routine blood smear at the clinical lab at SPH. Gordon Benson, a gastroenterologist at SPH was asked for his opinion on the slides because “someone thought I knew something about malaria”. Benson, after encountering no interest from the Massachusetts Department of Public Health, called the CDC on 16 July about a possible autochthonous malaria case. The key people in the story of the index case were thus a concerned family friend, an excellent clinical lab microscopist, and a phone call from someone who knew that malaria cases needed to be reported.

**Figure 3:**
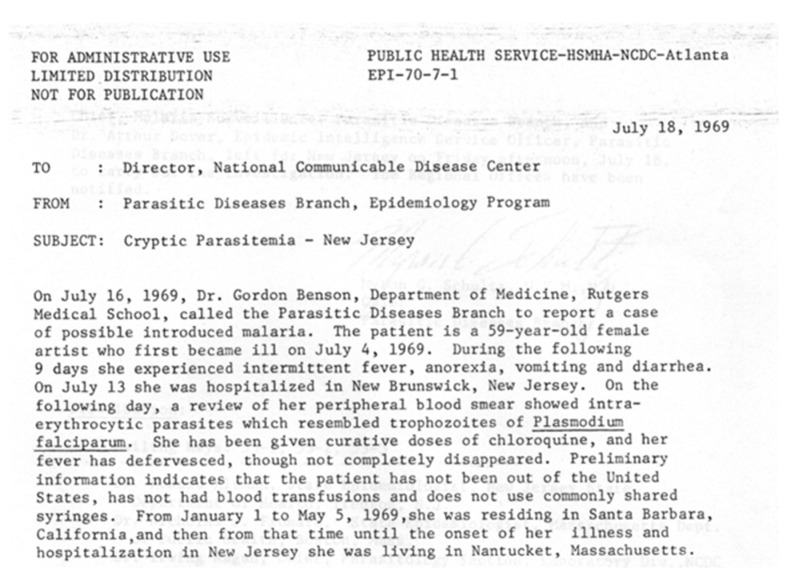
Internal CDC memorandum reporting the index case of babesiosis on Nantucket.

On 18 July, the CDC chief of malaria surveillance, Karl Western, and Arthur Dover, an Epidemic Intelligence Service (EIS) officer, left for New Jersey. The blood smears that prompted Dr. Benson’s call, arrived at CDC on 18 July. Mike Schultz, who was director of the parasitic diseases division of the CDC at the time, argued with their expert parasitology microscopist, Neva Gleason, about the identity of the infection, insisting that it was not malaria (M.G. Schultz, personal communication). Gleason found tetrads on her third reexamination of the slides and Schultz, who had earned both DVM and MD degrees and had trained at the London School of Hygiene and Tropical Medicine with the eminent malariologist Leonard Bruce-Chwatt, immediately knew this was a case of babesiosis. The internal National Communicable Disease Center memo, dated 18 July, was titled “Cryptic parasitemia—New Jersey” (Figure 3). The investigation and case report conducted by Western and Dover was published in the NEJM [1] almost verbatim from the internal 5 December 1969 CDC report, although Dover was surprisingly not included as an author.

Blood from the patient received on 19 July was subinoculated by George Healy, chief of the parasitology laboratory at CDC into hamsters and other experimental animals and propagated by subinoculation of hamsters [6]. The identity of the parasite was inferred from its morphology and infection kinetics in a prairie vole (*Microtus ochrogaster*) model and its comparison with *B. rodhaini* and “*B. microti*” from California rodents [7]. As was convention at the time (no longer done due to patient confidentiality), the parasite isolate was designated the Gray strain. Ristic et al. [8] also report isolation of *B. microti* from a blood sample taken from the index patient on August 4, with parasites passed into hamsters as well as a splenectomized monkey. The Gray strain was deposited at ATCC by the malariologist Julius Kreier at Ohio State. The provenance of the material that Kreier deposited (as outlined in the ATCC specification sheet) was “Western-Holbrook-Kreier” (presumably A. Holbrook of USDA, an expert on the ruminant babesias) and not from his frequent collaborator Miodrag Ristic (expert on diverse hemotropic infections, University of Illinois). As far as we know, the Gray strain has not been replenished from other sources in the interim, and what is available from ATCC (catalog number 30221) has been serially passaged between hamsters countless times from the original Kreier material. The ATCC material has been confirmed to have a Nantucket origin by variable nucleotide tandem repeat genotyping and by whole genome analysis [9,10].

The index case would have remained just the 5th sporadic case of babesiosis but in September of 1973, another case was diagnosed, this time at the Nantucket Cottage Hospital (NCH). A 50 year old female was admitted for fever and chills 25 days after she removed a tick; she had started having daily fevers 11 days afterwards but had symptomatic relief by the use of aspirin. The dates for these events and some details of her course of illness differ between the hospital discharge report and the case report [11], but the main features of the case are (1) the patient sustained daily or intermittent fevers to 104 °F that led her to seek medical attention; (2) that tetracycline (no dose indicated) was started because Rocky Mountain Spotted Fever was endemic on Cape Cod and the islands at that time; (3) that a blood smear was examined when the patient failed to improve on tetracycline; (4) that chloroquine phosphate 1.5 grams was provided in the first 24 hours after finding parasites on blood smear, then at 0.5 gram daily, presumably by mouth; (5) that she defervesced and felt better within 3 days of starting the chloroquine, despite parasites continuing to be found at low level in her blood smear; (6) Her hematocrit dropped to 23% on the sixth day of admission and she required two units of blood; (7) She was discharged on September 22 with no fever and was maintained on chloroquine twice weekly for 6 months. Interestingly, the case report mentions subinoculating two gerbils (“dictated by the availability of such animals in the local pet store”) with blood from the patient on the day parasites were found on blood smear, presumably by Dr. Anderson of Cape Cod Hospital, who was the consulting pathologist for NCH. Both gerbils became infected, and a second sample of blood, retained for 5 days in the refrigerator, infected hamsters at the CDC, which maintained the strain. This is the origin of the Peabody-mjr strain (ATCC PRA-99), which was adapted to inbred mice [12] by serial passage. C3H mice were found to be most susceptible in the first experiments trying to adapt hamster origin parasites. Subsequent work using the Peabody strain in balb/c mice confirmed earlier work with the British *B. microti* King’s strain [13] that the protective effect of adoptive transfer of immune splenocytes or lymph node cells was abrogated when the infected donor mouse had been depleted of T cells [14], suggesting a requirement for both T and B cells in controlling parasitemia.

Rodent subinoculation, as done by the CDC parasitologists and by Dr. Anderson, became the gold standard for confirming a case of *B. microti* babesiosis until the advent of polymerase chain reaction assays. Hamsters required only 300 parasites to become infected [15], which provided great sensitivity for confirming a diagnosis because one could intraperitoneally inoculate as much as 1 mL of blood (depending on the size of the hamster); the theoretical sensitivity was thus 0.3 parasites per microliter of blood, which is the same as a typical PCR assay using agarose gel detection of amplicons [16]. In practice, hamster inoculation could be complicated by transient parasitemias being missed or the hamster not being monitored a full month after subinoculation, and some parasites “preferred” splenectomized or immune-deficient mice and thus infection never became patent in hamsters (unpublished).

No cases were identified in 1974, but in 1975, an additional 6 cases were diagnosed on Nantucket, prompting another EIS investigation, this time headed by Trenton Ruebush II. The EIS report dated 9 January 1976 provided details on 6 cases, but the published report included only 5 of these [17]. The omitted case was a 86 year old male with fever, shaking chills, drenching sweats, myalgia/arthralgia, fatigue, splenomegaly, and hepatomegaly. A parasitemia of 25% was recorded on admission, but a blood smear taken 2 weeks previously was positive when retrospectively examined. The patient improved with chloroquine treatment, as did the other 5 cases, but he was parasitemic for another 10 weeks after discharge. This case was likely that alluded to in the first paper on reservoir hosts on Nantucket [18] as having been diagnosed in October. All but one of these first 8 cases from Nantucket were in people aged 50 years or older, establishing the fact that clinically apparent disease was associated with age.

A particularly interesting observation is that none of the early clinical reports [1,2,11,19] alluded to erythema migrans or other rash, although the first two cases sustained “tick bite” reactions, one of which resolved when the site of the bite was excised. About a fifth of babesiosis cases have evidence of concurrent Lyme disease [20] and a similar proportion of host seeking ticks on Nantucket infected by *B. microti* also contain *B. burgdorferi* [21]. However, an “insect bite” was reported in the first case from Shelter Island [22] and although the swelling that was reported is not typical for erythema migrans, the lesion abated with ampicillin treatment. Nonetheless, cases of odd rashes were not noted during the intensive EIS investigations of 1969 and 1975–1976, suggesting that *B. burgdorferi* was not commonly infecting people there at the time. This is a paradox, given that the agent had been enzootic in the area since the late 1890s [23] and certainly must have been co-transmitted by *I. dammini* long before human risk was apparent.

The early cases had been treated with chloroquine due to clinical similarity with malaria, but the efficacy of chloroquine had been questioned even for the index and subsequent cases. Parasitological cure was not demonstrated, and the drug had no effect whatever in reducing parasitemia in experimental hamster infections [24]. It should be noted, though, that in all of the early cases, prompt symptomatic relief was noted when chloroquine was provided, certainly well within the range of the week that was observed for symptomatic relief when current drug regimens (quinine/clindamycin or atovaquone/azithromycin) are used. Excess production of pro-inflammatory cytokines appears to be the basis for the signs and symptoms of acute babesiosis [25]. Chloroquine is immunomodulatory, inhibiting immune activation, including cytokine production [26] and would be expected to provide symptomatic relief. Although babesiacidal therapy is the standard, there may be benefits to immunomodulation in the treatment of babesiosis, and its possible role in combination therapy should be reexamined.

## 4. Epidemiology and Ecology of *B. microti* Babesiosis

Ruebush’s comprehensive epidemiologic investigation in 1975 included a home visit and telephone survey of the frequency of tick bites and febrile illnesses and a cross-sectional serosurvey. The results were published in 3 remarkable papers [17,19,27]. Complementary studies of potential reservoir hosts and vectors were done at the same time by his CDC colleague George Healy (who had undertaken the hamster subinoculation studies of the 1969 and 1973 cases) and Andrew Spielman of the Harvard School of Public Health [18,28]. The main findings were that 8.3% of 687 Nantucket residents (seasonal and full time) had sustained a tick bite in the 4 months preceding the survey; that asymptomatic infection was common (21 of 673 Nantucket residents were identified as seropositive, and of the 19 that could be followed up, 13 denied any febrile illness within 6 months of providing the blood sample). Ruebush et al. [19] made the prescient remark “Although transfusion-induced babesiosis has never been reported in man, the prolonged parasitemia noted in Case 3 suggests that transmission by this route may occur”. Babesiosis is now the most common protozoal hazard associated with blood transfusionin the United States [29].

On the ecology aspects, the team determined that 80% of white-footed mice (*Peromyscus leucopus*) trapped on Nantucket were infected. Spielman, using larval “*I. scapularis*” derived from engorged females removed from hunter killed deer on Nantucket, demonstrated vector competence by transmitting the Gray strain between hamsters. The ecology of babesiosis on Nantucket became the focus of Spielman’s graduate student, Joe Piesman, who developed methods of detecting *B. microti* in ticks (with the key finding that the parasites needed to be reactivated with the formation of sporozoites in order to be detected by microscopy), experimental challenge of deer (which were not susceptible), and seasonality of transmission [30].

Spielman and Piesman noted morphologic differences between the Nantucket “*I. scapularis*” and those from elsewhere in the eastern U.S., and described it as a new species, *Ixodes dammini*, with Nantucket as the type locality [31]. Although the name *I. dammini* was synonymized with *I. scapularis* in the early 1990s and few now use the junior subjective synonym, there are epidemiological reasons to continue to make the distinction, viz., the northern form bites people in the nymphal stage [32]. The main argument for synonymy was that ticks from southern sites (*I. scapularis*) would form fertile F1 hybrids with ticks from Massachusetts sites (*I. dammini*), but we now know that this is not a useful criterion to test for conspecificity in *Ixodes* ticks [33]. There are well defined genetic lineages of “*I. scapularis*” (likely a species complex) across the eastern U.S. [34] and it is likely that future analyses will reject the hypothesis that all the lineages have public health significance.

Gustave J. Dammin, after whom the tick was named, was pathologist in chief at the Peter Bent Brigham Hospital and part of the team undertaking the first successful kidney transplantation that won its surgeon, Francis Moore, a Nobel Prize in 1990. Dammin had married Anita Coffin, whose family were descended from the colonial founders of Nantucket in 1659, and frequently summered on Nantucket and nearby Tuckernuck Island. Dammin, who had made numerous notable contributions to tropical medicine and pathology, was keenly interested in the new infection, particularly after 1983 when he sustained an erythema migrans (perhaps the first well documented case on Nantucket) and made the rounds at the hospital whenever possible to tabulate babesiosis cases. He provided logistical assistance, funding, advice, and encouragement for Spielman’s early studies.

## 5. Has Risk Changed over 50 Years?

**Figure 4:**
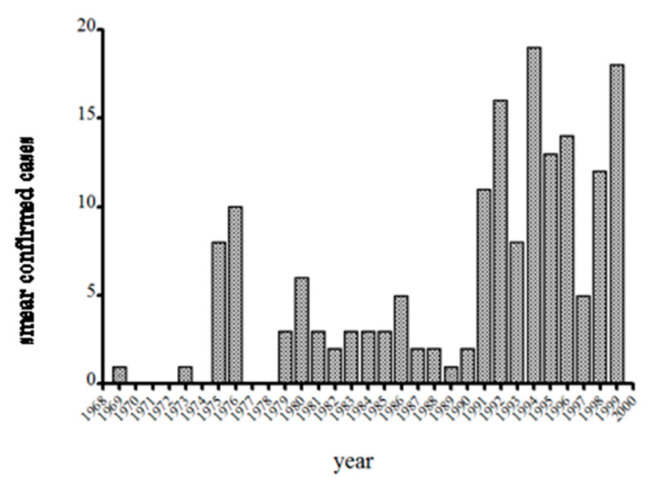
Nantucket cases identified from 1969-1999. All cases were blood smear positive. Cases from 1969–1986 compiled from the literature, or from Dr. G.J. Dammin’s notes. 1987–1999, compiled by T.J.L./S.R.T.III.

With confounding due to changes in the population at risk and enhanced awareness by local physicians, one might argue that it is difficult to test the hypothesis that risk has changed. The number of cases on Nantucket over the years fluctuates, ranging from none to a couple dozen, with a median of 13 from 1991–1999 (data collected by S.R.T.III and T.J.L.). One might argue that the sensitivity of identifying cases was probably great in the early years, when the infection was new and then as novelty waned, so did physician and laboratorian interest. However, the late Patricia Snow MT was the main microscopist at the Cottage Hospital from 1973 until the mid 1990s and indeed she was the microscopist for the second case [11]. Snow kept a log of all babesiosis cases, and with the arrival of T.J.L. in 1982, as well as Dr. Dammin’s efforts suggests reasonably good consistency in the level of effort taken to diagnosing babesiosis at NCH. Spielman gave S.R.T.III the responsibility of confirming cases by hamster inoculation, and reviewing all blood smears sent to him by Snow and this provided additional consistency to the data and thus comparable between years. The graph of annual babesiosis cases detected at NCH from 1969–1999 (Figure 4), then, is at least a consistent effort among years by the same people. The increase in cases detected from 1992–1997 does not reflect the advent of PCR or a change in risk. Snow had recently purchased a Coulter counter to perform complete blood counts and differential cells counts, and from her experience knew that babesiosis was accompanied by low platelet counts and leukopenia. She had the machine flag any such sample from a febrile case, and then spent 30 min on the microscope with the blood smear instead of the typical 10 min. This doubled the number of confirmed cases (with positive blood smear as the gold standard).

**Figure 5:**
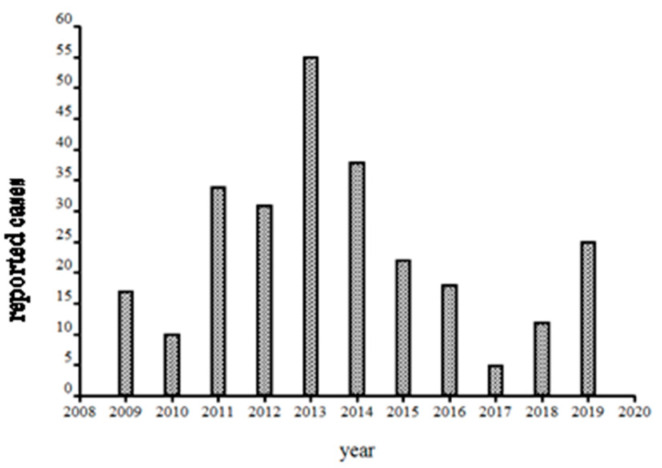
Nantucket cases reported to and by the Massachusetts Department of Public Health following mandatory direct laboratory reporting instituted in 2007. Data courtesy of Susan Soliva, MADPH Bureau of Infectious Disease and Laboratory Sciences.

**Figure 6:**
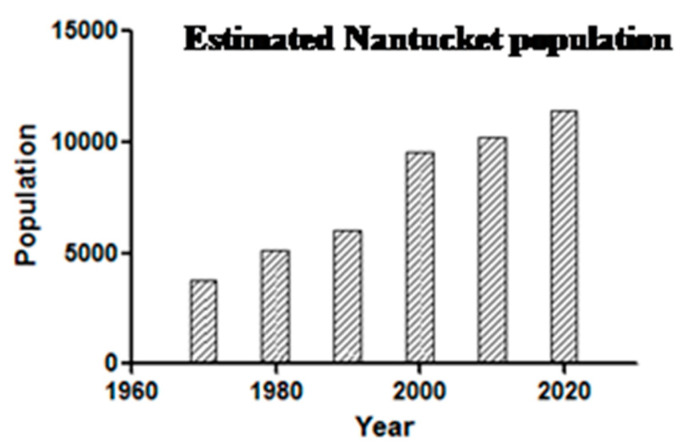
Doubling of Nantucket population 1970–2020. (Data from the Town of Nantucket; also available from www.nantucketdataplatform.com/projects, accessed on 30 July 2021).

**Figure 7:**
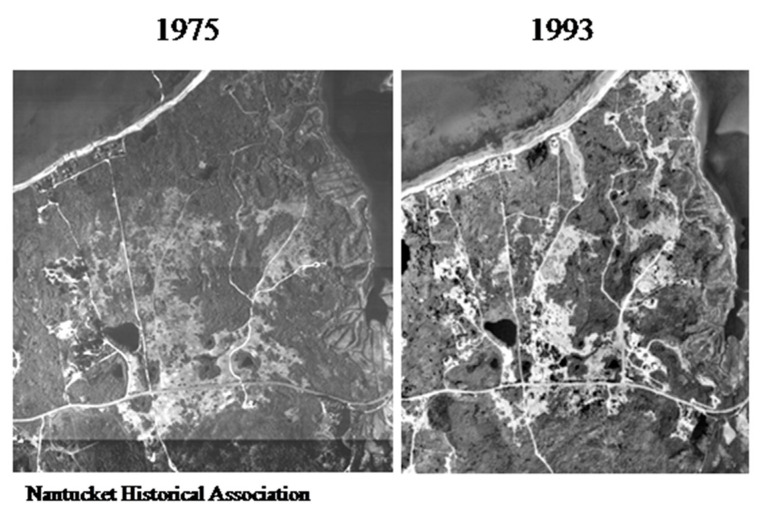
Increased development on Nantucket. Nantucket Historical Association, Aerial views of the Quaise/Polpis area demonstrating an increase in access roads and cleared spaces for new or larger houses; many older homes were purchased, razed, and new megamansion compounds built on the site. Source: https://www.nha.org/digitalexhibits/aerialviews/AP1975Web/index.htm; https://www.nha.org/digitalexhibits/aerialviews/AP1993Web/index.htm, accessed on 30 July 2021.

After 1999, the hospital changed from in house detection to a commercial laboratory (Imugen, Inc., which is no longer in existence) and thus estimates of case numbers are elusive. Massachusetts started mandatory reporting for babesiosis in 2006, with automatic electronic submission directly from commercial laboratories. Thus, the reports from 2009 onwards (oddly, fewer than 5 cases were reported from Nantucket during 2007 and 2008; the Nantucket Board of Health counted vastly more cases, personal communication to S.R.T.III and T.J.L.) are likely to be comparable between years (Figure 5). A median of 23 was reported from 2009–2019, suggesting a doubling in annual incidence from earlier years. Such a doubling likely reflects increased human exposure and susceptibility, not changes in the force of enzootic and zoonotic transmission: the population has doubled (Figure 6) and there has been a 12% increase in the number of Nantucket residents older than 65 years. Then, too, even though 40% of Nantucket land is held in perpetuity for conservation, thanks to the Nantucket Conservation, Land Bank, Massachusetts Audubon, and other organizations, the pace of development has greatly increased. The density of homes in buildable plots of land has increased over time; developers buy up older homes and subdivide the properties (Figure 7).

**Figure 8:**
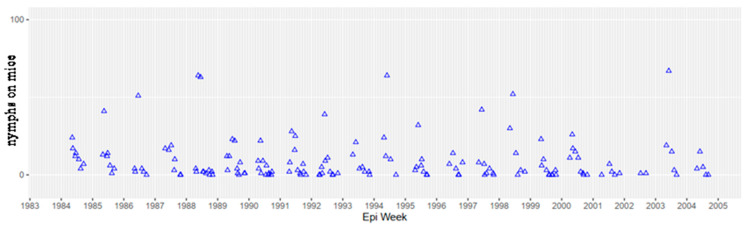
Nymphal *I. dammini* infestations on the UMass Field Station. Each datapoint represents the burden of an individual mouse. An intensive capture-mark-release study of *P. leucopus* was undertaken from 1984–2004, with monthly sampling from May to September of each year. No general trend (increasing or decreasing) is apparent, suggesting that the population has been at ecological equilibrium for 20 years.

There is no evidence for an increased force of enzootic or zoonotic *B. microti* transmission. The comprehensive studies in 1975–1976 by Ruebush and colleagues reported 3.1% (95% confidence interval, 2.0–4.8) seroprevalence in a cross-sectional serosurvey using NCH discard sera (samples taken for routine blood work). T.J.L. and S.R.T.III determined that 4.3% of 4524 (95% CI, 3.7–4.9) NCH discard sera from 1991–1997 were seropositive, using the identical indirect immunofluorescence assay [35] used by Ruebush, with the same cut-off (1:64 IgG). There was a median annual prevalence of 10% *B. microti* infection in host-seeking nymphal *I. dammini* from 1984–1991, estimated by dissection of salivary glands and Feulgen staining [21,36]; Telford unpublished, and the same median annual prevalence was observed from the same sampling sites during 2016–2021 using PCR (Goethert unpublished). The tick population was at equilibrium from 1985–2004 (Figure 8), as measured by indices of infestation of white footed mice. More recent trapping studies in the same Nantucket field sites (since 2005, trapped only during June and September, to provide annual indices of nymphal and larval *I. dammini*) do not indicate any differences in mouse infestation. Even though the force of transmission has not appreciably changed, there is evidence that genetic diversity of *B. microti* in ticks and mice has increased from 1987–2013, with more diversity of minor variable number tandem repeat (VNTR) genotypes in later years; samples from 1986–1988 were dominated by the 49e haplotype [37].

Nantucket was not the original source of American *B. microti* and did not seed the remainder of southern New England, despite all the cases of “Nantucket Fever”. There are 3 major lineages of *B. microti* across its range in the northeastern U.S. [37] suggesting that there were relict, epidemiologically silent enzootic foci across southern New England and that parasite range expansion was driven by local intensification and expansion into nearby areas. *B. microti* had been reported from rodents on nearby Martha’s Vineyard in 1937 [38], and cases of human babesiosis were identified from that island and eastern Long Island very quickly after the few Nantucket fever cases [22,24]. The peculiar focus in early zoonotic *B. microti* to the terminal moraine sites from Long Island to Cape Cod suggested that these were the first sites after the retreat of the Wisconsin glacier to be reinvaded from refugia farther south, serving as relict longstanding foci [39]. Consistent with this hypothesis, the upper midwestern zoonotic *B. microti* foci in Wisconsin and Minnesota are north of a prominent glacial refuge (“driftless zone”).

## 6. The Deer Tick Microbial Guild

Nantucket recorded its first cases of Lyme disease in the early 1980s, and the foundational babesiosis studies done by Spielman and Piesman on *I. dammini* and its ecology were immediately relevant to understanding risk for that bacterial zoonosis. *B. burgdorferi* was immediately incorporated into the existing babesiosis ecology and epidemiology program at Harvard. When human granulocytic ehrlichiosis (now “human anaplasmosis”) due to *Anaplasma phagocytophilum* was identified in northern Wisconsin [40], a prospective search was made by T.J.L. for febrile patients with elevated liver function tests, headache, and inclusions in their neutrophils. Pat Snow’s microscopy clinched the diagnosis of the index case for the northeastern U.S. [41] and led to the description of the agent’s natural history [42]. A focus on infections of cottontail rabbits identified a *Babesia divergens*-like parasite that was genetically identical to that causing MO-1 babesiosis, and the detailed ecological work [43] provides a basis for explaining its apparent rarity as a zoonosis; the vector is *I. dentatus*, which rarely bites humans. It remains a puzzle as to why MO-1 parasites have never been detected in febrile Nantucket residents despite determined efforts by T.J.L., S.R.T.III and H.K.G. When deer tick virus (Powassan lineage II) was discovered in 1995 [44], a virus isolate (NFS001) was quickly made from Nantucket ticks and evidence of *P. leucopus* exposure there demonstrated [45]. Again, it is a puzzle why a deer tick virus encephalitis case has never been detected from Nantucket given its regular detection in host seeking *I. dammini* there over the years, despite intensive efforts by T.J.L. and S.R.T.III.

## 7. Control and Prevention

From the very beginning, CDC and Harvard investigators attempted to make recommendations for risk reduction. In the 9 January 1976 EIS report, the concluding paragraph made points that are still germane today. “Temporary and permanent residents of the island and medical personnel should be alerted to the risk of a tick bite and the characteristic symptoms of babesiosis … Before control programs directed against vectors or reservoir hosts of babesiosis can even be considered, studies are needed to identify and define: (a) other possible tick vectors; (b) additional reservoir hosts in wild and domestic animals; (c) the distribution and fluctuation in the vector and reservoir host populations, and (d) the prevalence of infection in vector and reservoir hosts. Finally the risk of infection for man must be determined so that a cost-benefit analysis can be made of proposed control measures”. The Nantucket Board of Selectmen was more to the point; Andy Spielman spoke of a grizzled seaman with tattooed arms asking him “so, doc, what do we spray?” Even though we know the main aspects of *B. microti* perpetuation, its reservoirs, prevalence of animal and tick infection, and the risk of infection, no cost-benefit analysis has been done and no sustained public health effort has been made to reduce the risk of babesiosis or Lyme disease.

Nantucket served as the control site for the seminal Great Island deer reduction experiment [46] and deer reduction has been strongly advocated for Nantucket, first by Spielman and subsequently by S.R.T.III and T.J.L. However, reducing the deer herd is hindered by sociopolitical factors that include a public reluctance to kill Bambi (the embodiment of charismatic megafauna). Habitat management is difficult: an attempt by S.R.T.III to convince Nantucket landscapers to propose to homeowners that vegetation be eliminated around their homes (thereby removing microhabitat needed for the tick to survive) was met with derision. “People want that thick stuff there for privacy”. Damminix tick tubes [47] remain available but few homes regularly and consistently use them. “Spraying” is effectively prohibited as a mode of intervention due to the fact that all of Nantucket’s freshwater comes from a freshwater “lens” 40–500 meters below the ground surface and people are concerned about chemicals contaminating it. Personal protection remains the best preventive method, which comprises promoting awareness, using repellents, permethrin treated clothing, and doing tick checks. The late Jim Lentowski, director of the Nantucket Conservation Foundation for 40 years, was the island leader in promoting awareness, having suffered from 3 of the 5 deer tick-transmitted infections.

The hopes for vaccination remain doubtful, particularly given the general failures (even with tremendous effort and funding) for effective vaccination against the related malarial parasites. Even if an effective and safe vaccine were developed, market analysis by pharmaceutical companies would not support the investment of $150 million or more to acquire the data for U.S. Food and Drug Administration approval. Indeed, Nantucket was one of the sites for Phase II and Phase III trials of Lymerix [the human Lyme disease vaccine developed by SmithKline Beecham (King of Prussia, PA, USA) and approved by the Food and Drug Administration], a safe and effective product that was sold for 5 years and then withdrawn from the market because sufficient cost recovery was not apparent in the face of anti-vaccine activism. Even an effective vaccine has no guarantee of financial and public health success. It is discouraging that after 50 years of research, we seem to have done little to reduce the risk for acquiring babesiosis on Nantucket.

One success in developing interventions in the last 50 years is that of therapy for babesiosis. Nantucket was the main site for the pivotal prospective randomized clinical trial of atovaquone and azithromycin (AA) [48]. Clindamycin and quinine (CQ), which had been demonstrated effective for parasitological cure in hamster infections [49] had been the treatment of choice for *B. microti* babesiosis since 1983 [50]. In the clinical trial, AA induced a more rapid parasitological response than did CQ, and did so with only 15% of the subjects reporting adverse reactions to treatment, compared with 72% for CQ. However, azithromycin, clindamycin, and quinine have failed to clear parasitemia in mouse models of *B. microti* infection [51], and treatment failures are not unusual, particularly in immunocompromised patients. Additional drug regimens for treating babesiosis are needed, particularly for those who are immunocompromised [52].

## 8. What Can We Predict for Nantucket Fever at Its Centennial?

In the ideal future world, a common sense approach will be taken on environmental modification with combinations of habitat management, environmentally friendly, targeted insecticides, robotic vector control, and deer reduction. The landscape is successional; humans have influenced every inch of Nantucket land and there should be no protected worship of poison ivy and invasive plant-dominated habitat. A return to pasture and heathlands would reduce habitat for deer ticks and white footed mice. Deer reduction must be strongly pursued, even if an effective and economical sterilization method was available (reduce the herd, then control their reproduction). Mechanical means of removing host-seeking deer ticks might be accomplished by advances in robotics; future generation insecticides might be developed with less impact on non-target species or more degradable to preempt suggestions of drinking water contamination.

In 2069, genetic modification of reservoir hosts and ticks and replacement of their populations with those genetically modified to be less competent to maintain enzootic transmission will no longer be considered science fiction nor evoke suspicion. Indeed, efforts are underway to modify *P. leucopus* so that they constitutively and heritably express anti-OspA, rendering them less reservoir competent for *B. burgdorferi.* Nantucket is a candidate site for larger field trials to release such mice, once small scale proof of concept regulatory studies have been completed [53]. *B. microti* antigens that appear to induce some degree of transmission blocking immunity could easily be incorporated in such a platform.

Anti-tick vaccines, which would reduce the risk for the transmission of all 5 of the deer tick transmitted zoonoses (in the northeastern U.S.; a sixth, *Ehrlichia muris*, is present in the upper midwestern states) by interfering with tick feeding, may become available and could be commercially successful. When Lymerix was deployed, there was debate on its merit because the vaccine only protected against Lyme disease and people would still have to take all required precautions to avoid infection with the other deer tick-transmitted pathogens. Accordingly, an anti-tick vaccine could be more widely acceptable. The current situation with COVID-19, however, demonstrates that even when an effective vaccine is available, people may not avail themselves of its benefits; accordingly, environmental approaches remain critical to develop and implement.

Personal protection remains the only prevention method that could greatly reduce risk at the individual level if consistently practiced. Permethrin treated clothing is now readily available from online retailers at prices that should not deter their routine use. Joe Piesman, who saw the beginnings of the zoonotic situation that has intensified across the eastern U.S., frequently said in his role as Lyme disease vector studies chief at CDC, “we know what we have to do, we just can’t get people to do it”. Alas, the same may be said about any public health issue, from smoking to heart disease to sexually transmitted infections.

Even if a new Nantucket fever appears in the next 50 years (*ex Nantucket semper aliquid novi*) we remain optimistic that the technological advances that will have taken place by 2069 will provide the great public health benefits for Nantucket residents and visitors that unfortunately have eluded us in the last 50 years despite a strong evidence basis for diverse interventions.

## Data Availability

Not applicable.
